# DVFNet: A deep feature fusion-based model for the multiclassification of skin cancer utilizing dermoscopy images

**DOI:** 10.1371/journal.pone.0297667

**Published:** 2024-03-20

**Authors:** Ahmad Naeem, Tayyaba Anees

**Affiliations:** 1 Department of Computer Science, School of Systems and Technology, University of Management and Technology, Lahore, Pakistan; 2 Department of Software Engineering, School of Systems and Technology, University of Management and Technology, Lahore, Pakistan; National Textile University, PAKISTAN

## Abstract

Skin cancer is a common cancer affecting millions of people annually. Skin cells inside the body that grow in unusual patterns are a sign of this invasive disease. The cells then spread to other organs and tissues through the lymph nodes and destroy them. Lifestyle changes and increased solar exposure contribute to the rise in the incidence of skin cancer. Early identification and staging are essential due to the high mortality rate associated with skin cancer. In this study, we presented a deep learning-based method named DVFNet for the detection of skin cancer from dermoscopy images. To detect skin cancer images are pre-processed using anisotropic diffusion methods to remove artifacts and noise which enhances the quality of images. A combination of the VGG19 architecture and the Histogram of Oriented Gradients (HOG) is used in this research for discriminative feature extraction. SMOTE Tomek is used to resolve the problem of imbalanced images in the multiple classes of the publicly available ISIC 2019 dataset. This study utilizes segmentation to pinpoint areas of significantly damaged skin cells. A feature vector map is created by combining the features of HOG and VGG19. Multiclassification is accomplished by CNN using feature vector maps. DVFNet achieves an accuracy of 98.32% on the ISIC 2019 dataset. Analysis of variance (ANOVA) statistical test is used to validate the model’s accuracy. Healthcare experts utilize the DVFNet model to detect skin cancer at an early clinical stage.

## 1. Introduction

Skin cancer arises from the unregulated growth of skin cells resulting in the development of malignancies [1]. Ultraviolet radiation exposure without protection is considered the major cause of these malignancies [2–11]. Melanomas comprise only 5% of all cases of malignant skin growths, whereas basal cell carcinoma and squamous cell carcinoma account for the remaining 95%. Squamous cell carcinoma [4, 8] is one of the diseases with the highest incidence rate and the most severe consequences in the United States. In the US, there are around 5 million cases of skin diseases registered over a single year [5, 10]. During the last few decades, there has been a steady increase in the incidence of skin cancer [12]. Every year, around 18,000 people are found to have invasive malignant melanoma for the first time [6].

In the United States, skin cancers account for 4% of all malignant neoplasms and roughly 1% of all cancer deaths are attributable to this form of the disease [13]. Despite this, there is a significant chance of survival from skin cancer when it is caught at an early stage [14]. From 2009 to 2019, the number of skin cancer cases examined annually increased by 55%, according to the currently available records [15]. It is anticipated that there will be an increase in the number of fatalities caused by this disease during the next decade [16]. The early diagnosis plays a vital role in the patient’s survival [17]. Detection at a later time reduces the chance of survival to less than 14%, whereas the chance of survival is greater than 90% if detected at an earlier stage [8, 18]. According to research, finding skin tumors is the most effective method for diagnosing skin cancer as soon as possible. Almost 16% of individuals with dermatological disorders have benign skin cancer, 3.3% have actinic keratosis and 3% have malignant skin cancers. This indicates that 20% of patients a medical professional examined were identified with a tumor [18] whereas, 11.4% of people have benign skin tumors, while only 5% have malignant skin cancers [7].

Generally, an expert dermatologist visually examines the suspected malignancies, followed by a dermoscopy and a biopsy [13–21]. The detection of skin cancer depends upon the skill of the dermatologist. Manual skin cancer detection is a laborious and time-consuming process for patients [10]. The level of knowledge possessed by physicians and the variety of dermoscopy methods in which they have been professionally educated might also impact the sort of diagnosis rendered [22, 23]. Consequently, numerous medical professionals hold contrasting views regarding the outlook for a specific illness.

Significant solutions are required to promptly diagnose skin cancer in its initial stages and find remedies for some of the aforementioned instances. These solutions were developed by improving the algorithms used for computerized image analysis [24]. The majority of these algorithmic definitions include parametric expressions [25]. Artificial intelligence (AI) emerges as a highly developed technology with a wide range of practical applications, as evidenced by recent advancements in the field [26–28]. Machine learning (ML) is an advanced field of study within AI that empowers computers to learn from and adapt to data patterns, surpassing the ability of computers to simply execute commands. Predictive algorithms are generated through ML by utilizing data observations to forecast future output. The ML is designed to implement a system that can independently acquire knowledge without requiring human intervention [29–31]. The performance of these ML-based algorithms suffers due to heterogeneous data, low-quality medical data, imbalanced datasets and irrelevant performance metrics of data [32]. Deep learning (DL) is one of the ML subfields that is expanding quickly [33]. DL is widely utilized in numerous fields such as covid 19 detection [34], heart disease detection [35], brain tumor detection [36], and dengue detection [37].

Therefore, the main focus of the researchers is to create a DL-driven diagnostic system that is capable of accurately identifying and classifying various forms of skin cancer in its initial phases [38]. Furthermore, by using deep learning and machine learning techniques, doctors can identify skin issues early, eliminating unnecessary surgeries and biopsies [39].

In this study, we introduced a unique multiple-categorization model based on the CNN for detecting Melanoma (MEL), Basal cell carcinoma (BCC), Squamous cell carcinoma (SCC), Actinic keratosis (AK), Benign keratosis (BK), Vascular lesion (VASC), Melanocytic nevus (MN), Dermatofibroma (DK) and others in dermoscopic images.

This research proposes a deep technique using Convolutional Neural Networks (CNNs) to identify cases of multiple types of skin cancers using dermoscopy image scans. This study made use of a publicly accessible research dataset from ISIC 2019. The data set contains 25,331 dermoscopy images of multiple types, such as MEL, AK, BK, DF, BCC, MN, VASC, and SCC. Our study also anticipates significant application of the feature extraction method. In this context, we present a model based on a VGG19 and the Histogram of Oriented Gradients (HOG) concept. Our suggested approach cleans The dermoscopy images from the artifacts using anisotropic diffusion. Segmentation is applied to extract the region containing the lesion on an image. The next step is to extract features independently using HOG and VGG19 and make the feature vector using feature fusion. This feature vector is applied to a convolution neural network (CNN) for multiclassification.

The following is a list of the utmost important contributions made by this study:

- The proposed model is introduced to detect eight distinct forms of skin cancer. The presented model extracts prominent features from dermoscopy images using HOG and VGG19, which aids in accurately diagnosing the disease.
- Modified Anisotropic Diffusion Filtering (MADF) removes the noise from the dermoscopy images. Whereas, segmentation is applied to extract the region containing the lesion on an image.
- To make a robust classifier, the trainable parameters are limited in this study to overcome the issue of model complexity.
- CNN models suffer from accuracy issues due to class imbalance in healthcare data. To solve this problem, we use the SMOTE Tomek up-sampling method, which gathers multiple samples from an image belonging to each class.
- The Grad-CAM heat-map method shows the prominent characteristics collected from the different techniques for classifying multiple types of skin cancer.
- Using a variety of assessment measures, including accuracy, precision, AUC, loss, recall, and F1 score, the proposed model outperformed four CNN baseline classifiers, such as VGG-16, ResNet-50, Inception-V3, and AlexNet.
- In addition, DVFNet performed better when compared with current state-of-the-art models.
- Analysis of variance (ANOVA) statistical test is used to validate the model’s accuracy.

This study is split into the sections listed below: In Section 2, we will discuss the available literature. The Methods and materials are covered in the Section 3. The discussion and results are presented in Section 4. The limitation of study is presented in the section 5. This investigation concludes in Section 6.

## 2. Literature review

Professionals who are well-versed in the technologies and applications of multimedia. In addition, they emphasized that the development of inexpensive methods for identification, including AI, might alter the testing routes of patients, allowing for more efficient medical care delivery [40]. In recent years, research and development have been conducted to create various deep-learning strategies for detecting, segmenting, and classifying. Several researchers have carried out a series of research that are connected. In their study, ConvNet is presented by Nahata and Singh [41] to categorize more than 35,000 photos from the ISIC dataset between 2018 and 2019. They use a variety of classification models.

Bansal et al. [42] applied three distinct morphological approaches to de-haring dermoscopy images. Since the quality of features influences the performance of a classifier, this study integrated features extracted from dermoscopic images by employing handcrafted (HC) and deep learning models (DLM). The extraction of features is performed by the ResNet50V2 and EfficientNet-B0. In comparison, artificial neural networks (ANN) are utilized for classification. The proposed method is evaluated using the PH2 dataset, which consists of 200 dermoscopic images with 40 melanoma and 160 non-melanoma images, and the HAM10000 dataset, which is part of the ISIC 2018 challenge. On the HAM10000 and PH2 datasets, the proposed model achieves 94.9% and 98.0% accuracy, respectively. Shetty et al. [43] proposed a model that applies data augmentation on the dataset to enhance the model’s precision. This work uses the k-fold cross-validation method to guarantee the model’s robustness. CNN models and ML techniques were employed to evaluate the classification accuracy. The study’s findings showed that the suggested CNN outperforms other popular techniques in terms of accuracy. The model has the maximum overall accuracy of 95.18% on the HAM10000 dataset.

To increase effectiveness and performance, Iqbal et al. [44] employ a DCNN model with several layers, filters and finetuned parameters. The performance of the suggested technique is verified using the ISIC 2017 dataset. Several performance matrics such as sensitivity, specificity, and accuracy verified the efficacy of DCNN approach. Using ISIC 2017, this technique obtains 94% accuracy, 93% recall, and 91% specificity. Karar et al. [25] constructed an image preparation pipeline. To make the dataset more suitable for the different models, they cleaned the photos by eliminating stray hairs and reduced the sizes of the photographs. The HAM10000 dataset is used to verify the efficacy of different models. Among the models EfficientNet B4 achieved the best accuracy of 87.91%.

Tang et al. [45] solve a long-standing problem by introducing a brand-new approach to segmenting skin lesions. The Adaptive Feature Learning Network (AFLN) is used with dermoscopy images to train feature representations. Incorporating multi-scale data into the AFLN model was made possible by developing a fusion model using an ensemble learning method. This model resolves the issues of overfitting by utilizing DGCL. ISIC 2016, 2017 and 2018 are utilized to determine the model’s performance. The model achieves an accuracy of 93.10% on ISIC 2016, 87.50% on ISIC 2017 and 96.60% on ISIC 2018. Panthakkan et al. [46] proposed a concatenated Xception-ResNet50 (X-R50) model that classifies various skin malignancies. The effectiveness of the proposed strategy is compared to innovative transfer learning techniques. Deep CNN is used as a standard to examine other models. The HAM10000 dataset was employed to evaluate the efficacy of the suggested model. In this study, 10,000 photos of the epidermis were utilized. Sliding window analysis is used for both training and testing of the model. The predicted accuracy of the suggested X-R50 model is 97.8%.

Mijwil et al. [47] selected and trained a deep-learning network to analyze over 24,000 skin cancer images. The ConvNet model used three distinct architectures: ResNet, VGG19 and InceptionV3. The results were excellent; by employing many factors, this study designed optimal solutions for the binary classification as benign or malignant. The proposed model achieves an accuracy of 86.90%. Qasim et al. [48] used the surrogate gradient descent method to preprocess the images of the ISIC 2019 dataset, which consists of 3323 non-melanoma images and 3670 melanoma images, using deep spiking neural networks. The suggested spiking VGG-13 model achieved an accuracy of 90.07% and F1 score to 90.07% and 89.57%, respectively.

Dong et al. [49] present a new Cross-Modal Collaborative Feature Exploration (CMC) tool that lets people work together to look for hidden features in dermoscopic images. Diverse interaction effects among cross-modal features are dynamically captured by the CMC module during the learning phase of the model. With the best segmentation model’s lesion masks, we crop the images to improve the classification ability by distinguishing between lesions. The proposed algorithm is tested on four publicly accessible datasets of skin utilized for lesions: ISIC 2018 and PH2 for segmentation, whereas ISIC 2019 and 2020 were used for classification. This model obtains a superior accuracy of 92.6%. Tembhurne et al. [50] proposed a new method for identifying skin cancer employing a hybrid method. The ML model analyzes features using methods like the contourlet transform and the local binary pattern histogram, the deep learning model employs revolutionary neural networks. Any image classification problem must be solved by feature extraction with meaningful significance. Because it integrates handcrafted and automated elements, the model performs better than previous ones; it has a 93% accuracy for binary disease classification.

He et al. [51] employed a co-attention fusion network (CAFNet) that had a hyper-branch for continuous boosting and fusing and two subnets for identifying dermoscopy and clinical features. The hyper-branch consists of many co-attentions fusion (CAF) modules. To facilitate cooperation across several senses, it is necessary to first develop a CA block that incorporates a cross-modal attention system into each CAF module. By allowing the two modalities to interact with one another, this block increases the representational power of the extracted features. The CA block is then followed by an attention fusion (AF) block, which fine-tunes the fusion properties by dynamically selecting the ideal fusion ratios for the pixel-wise multimodal fusion. A deep-supervised loss coupled with a mixed prediction strategy may provide the best results. CAFNet accomplished an accuracy of 76.8% on the 7-point dataset. Razmjooy et al. [52], created a method that uses the Multi-Agent Fuzzy Buzzard Algorithm (MAFBUZO) to enhance a histogram-based, multi-level optimal thresholding segmentation approach. The MAFBUZO algorithm combines the local search agents of MAFs with the global search capacity of the BUZO algorithm to find a happy medium between the exploitation and exploration stages of the optimization process. The Dermquest and DermIS databases are utilized to evaluate the technique’s efficacy. The overall accuracy of the model is 94%.

Omeroglu et al. [53] proposed a network consisting of multiple branches and a hybrid integration of feature maps from multiple modalities. These branches allow us to dissect multimodal interactions and draw out specific details. A soft attention module, a tweaked Xception architecture, and a specialized feature extraction method all work together to train the network to focus on individual skin lesions. The suggested framework was evaluated using a seven-point dataset that is accessible to the public. When utilizing numerous labels to diagnose skin lesions, this method attains an overall accuracy rate of 83.04%.

Ding et al. [54] presents a new approach for melanoma identification. To improve the quality of the input images, the suggested method normalize the data in the initial phase. In the next phase Gray-Level Co-occurrence Matrix (GLCM) is used to locate the different image attributes. The texture information for the images is gathered by GLCM. Support Vector Machine (SVM) is then used to classify the chosen features to differentiate between benign and malignant tumors. The American Cancer Society (ACS) dataset is used to validate the suggested approach. The suggested approach outperforms all other strategies that were examined in terms of each performance metric. The suggested method successfully diagnoses melanoma with 88% accuracy.

Viknesh et al. [55] provides a computer-aided detection method for melanoma early diagnosis and therapy. Two methods are suggested in this work for the detection of skin cancer. In the first technique, transfer learning methods, such as the AlexNet, LeNet, and VGG-16 models are used for the diagnosis of skin cancer. Furthermore, they investigate how different dataset sizes relate to the model’s depth and performance. The second method uses support vector machines to binary classify images. The SVM classifier retained a 91% accuracy, whereas the CNN achieved an accuracy of 86.6%.

To improve accuracy, Dahou et al [56] present a powerful skin cancer detection system that extracts and trains the system using a MobileNetV3 architecture. The acquired attributes are then put into an improved optimization algorithm named Hunger Games Search (HGS). To identify which feature is more relevant and to maximize the performance of the model. The developed DOLHGS was evaluated using the three-category PH2 dataset and the two-category ISIC-2016 dataset. The recommended model’s accuracy on PH2 is 96.43%, whereas it is 88.19% on the ISIC-2016 dataset. The results of the testing showed that when compared to other popular algorithms, the proposed technique had better optimized features and classification accuracy for skin cancer detection.

The ISIC 2019 dataset was utilized by Qasim et al [48] to classify 3323 non-melanoma photos and 3670 melanoma photos using the surrogate gradient descent technique and deep spiking neural networks. With less trainable parameters, the suggested spiking VGG-13 model beats the VGG-13 and AlexNet, obtaining an accuracy of 89.57% and an F1 score of 90.07%.

Mridha et al [57] created a precise deep learning (DL) approach particularly for the diagnosis of skin cancer. The primary reason for the class imbalance is frequently arises from the skin-affected patients because many disease classes are significantly smaller than the other classes. These imbalance classes affects the overall performance of models. Conducting a more thorough evaluation of the model’s output enabled a deeper understanding of the reasoning behind the decision. The researchers employed a CNN and the HAM10000 dataset to distinguish between the seven distinct varieties of skin cancer. The model was trained utilizing three activation functions (Relu, Swish, and Tanh) and two optimization functions (Adam and RMSprop). By achieving a loss accuracy of 0.47% and a classification accuracy of 82%, this approach exhibits promise in aiding medical practitioners in the proactive identification of skin cancer. Summary of literature review is presented in the Table 1.

**Table 1:** Literature review for the detection of skin cancer.

Ref	Year	Datasets	Classification	Model	Accuracy
[42]	2022	ISIC 2018	Actinic Keratosis, Benign keratosis lesion, Basal cell carcinoma, Dermatofibroma, Melanoma, Vascular lesion and Melanocytic nevus.	HC+DLM	94.9%
[43]	2022	HAM10000	Actinic Keratosis, Benign keratosis lesion, Basal cell carcinoma, Dermatofibroma, Melanoma, Vascular lesion and Melanocytic nevus.	CNN	95.18%
[44]	2022	ISIC 2017	Melanoma and Non-Melanoma	DCNN	94%
[25]	2023	HAM10000	Actinic Keratosis, Benign keratosis lesion, Basal cell carcinoma, Dermatofibroma, Melanoma, Vascular lesion and Melanocytic nevus.	EfficientNet B4	87.91%
[45]	2021	ISIC 2016, ISIC 2017 ISIC 2018	Melanoma and Non-Melanoma	AFLN +DGCL	93.10%
					87.50%
					96.60%
[46]	2022	HAM10000	Actinic Keratosis, Benign keratosis lesion, Basal cell carcinoma, Dermatofibroma, Melanoma, Vascular lesion and Melanocytic nevus.	X-R50	97.8%
[47]	2021	24000 skin cancer images	Melanoma and Non-Melanoma	ConVNet	86.90%
[48]	2023	ISIC 2019	Melanoma and Non-Melanoma	Spiking Vgg 13	90.07%
[49]	2023	ISIC 2020	Melanoma and Non-Melanoma	CMC	92.6%
[50]	2023	ISIC Archive	Melanoma and Non-Melanoma	HC+CNN	93%
[51]	2023	Seven-point dataset	Melanoma and Non-Melanoma	CAFNet	76.8
[52]	2023	Dermquest + DermIS	Melanoma and Non-Melanoma	MAFBUZO	94%
[53]	2023	Seven-point dataset	Melanoma and Non-Melanoma	Xception	83.04%
[54]	2023	ACS dataset	Melanoma and Non-Melanoma	GLCM+SVM	88%
[55]	2023	ISIC archive	Melanoma and Non-Melanoma	SVM	91%
[56]	2023	ISIC 2016 PH2	Melanoma and Non-Melanoma	MobileNetV3 + HGS	88.19%
					96.43%
[48]	2023	ISIC 2019	Melanoma and Non-Melanoma	Spiking neural networks	89.57%
[57]	2023	HAM1000	Actinic Keratosis, Benign keratosis lesion, Basal cell carcinoma, Dermatofibroma, Melanoma, Vascular lesion and Melanocytic nevus.	CNN	82%

Skin cancer detection is complicated due to the wide variety of images. Skin cancer is harder to find because people have a lot of different skin tones. Following the foremost challenges.

- The various sizes and forms of images made it hard to classify skin cancer. Therefore, preprocessing is essential for reliable analysis.
- To get the desired result, it is necessary to remove information that is not originally part of the image. As a result, abnormalities and noise must be filtered out before the actual processing begins.
- Poor contrast from surrounding tissues makes it difficult to diagnose skin cancer effectively.
- Color illumination creates difficulties in the detection of skin cancer.
- Some lesions on the human body might never develop into cancerous cells, but they make it challenging to detect skin cancer from malignant images accurately.
- Another difficulty in detecting skin cancer is the bias that hinders existing models’ effectiveness.

The death rates caused by malignant skin cancer are incredibly high. However, if it is detected and treated early, the patients have a higher chance of survival [39–44]. This motivates us to develop a skin cancer detection technology since every life is valuable and must be protected at all costs.

## 3. Proposed methodology

The proposed method is evaluated on the ISIC 2019 dataset that can be accessed freely and only deals with dermoscopic image collections of eight distinct types of skin cancer. Dermoscopy is an extensively utilized imaging procedure that enables the outermost layer of skin to be depicted by light-amplification via immersion fluid [1]; even so, dermoscopy’s diagnostic accuracy relies on dermatologists’ expertise. This study employed only publicly available datasets and presented an innovative technique for detecting skin cancer in dermoscopic images.

This section explains the proposed DVFNet model approach for identifying skin cancer on dermoscopic photos. The DVFNet was trained using dermoscopic images of various types of skin cancer, such as actinic keratosis (AK), basal cell carcinoma (BCC), benign keratosis (BK), dermatofibroma (DF), melanoma (MEL), melanocytic nevus (MN), squamous cell carcinoma (SCC), and vascular lesion (VASC). The sample data used in the training phase were highly qualified and diverse. To discover a Region of Interest (ROI), all input datasets are first converted to grayscale and then any undesirable regions are deleted. After accumulating patches derived from the ROI images, filtering and segmentation procedures are carried out. We additionally implemented a strategy named the synthetic minority oversampling technique (SMOTE TOMEK) to address the problem of an uneven allocation of databases and to equalize the amount of data points inside every category [8]. Integrating the VGG19 and HOG results in the production of feature vectors, which can then be used for feature extraction.

**Fig 1:**
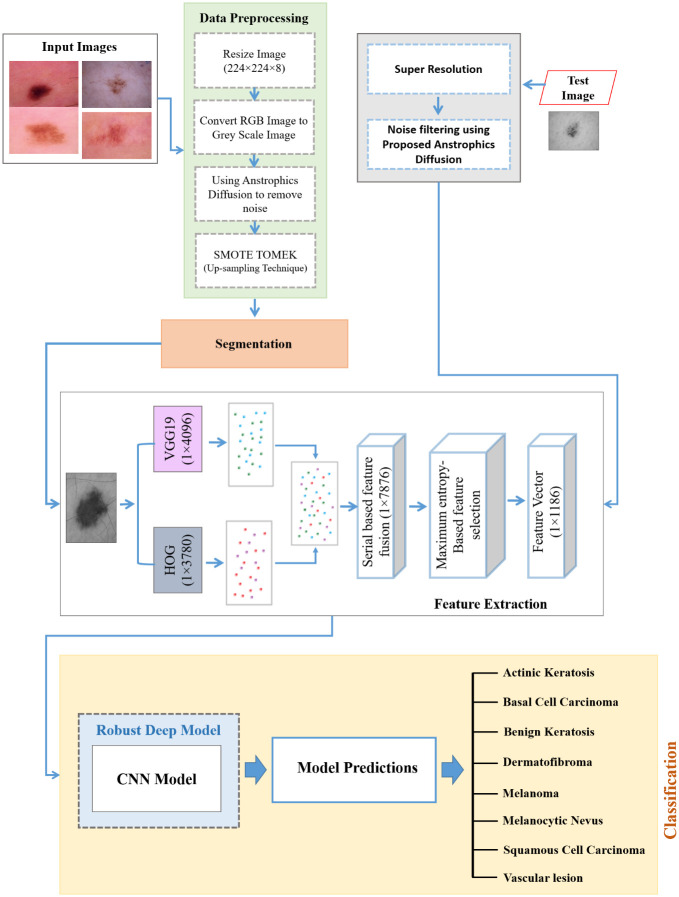
An outline of the workflow for the proposed DVFNet model.

Classification is accomplished by utilizing these feature vectors throughout the process. The method of the proposed system that works is illustrated in Fig 1 and expressed in Algorithm 1. The dimension of the input image has been set at 224 by 224 pixels. In our study, forty epochs are applied to train the model. After fulfilling all epochs, the proposed model had reached the accuracy level anticipated throughout training. This model was used to construct strategies for pre-processing and retrieval of features by utilizing VGG19, which has a complexity of O(n) for each training image. The HOG feature extraction approach with an O(n) complexity level has been applied to each image. When applied to an input image of n pixels, this model has a complexity of [O(n) + O(n)]. The Grad-CAM heat-map strategy was developed to clarify the apparent skin cancer characteristics contributing to the disease’s classification. These characteristics were utilized to emphasize the factors that are beneficial in the detection of skin cancer.

**Algorithm 1: Proposed DVFNet for Skin Cancer Diagnosis**

**Input**: Dermoscopic image dataset (T) of Skin cancer impacted patients using rescaling image (Q)

**Extraction**: Matrix for Feature Extractor (f)

**Start**

**Feature Vector of VGG19 (G**_**v**_ **)**

 1. Initialize G_v_ = > Q_j_. j = 1

 2. Feature Extraction of each image T (j, 1, 570)

 3. G_v_ (j, 1) = Q (x, 1). + G_v_ (j, 1)

 4. G_v_ = Entire Features of CNN

**Histogram of Oriented Gradients**

 1. Initialize D_0_ and D_1_, Where D_0_ is low pass output and D_1_ is bandpass output

 2. HOG (j, 1) = D_0_ (j, 1) + D_1_ (j, 1)

 3. HOG = Overall Histogram Oriented Gradient

 Feature Fusion in Vector (V).

 Training Vector V = | G_v_, HOG |

 test_img = read (img).

 Feature Extraction (FE) = step repeat 1, 2 from test_img.

 result (j) = predict (V, FE).

 Output: result (j) = AK, BCC, BK, DF, MEL, MN, SCC or VASC.

 End

### 3.1. Dataset description

The proposed model was developed and evaluated using a dataset derived from the International Skin Imaging Collaboration (ISIC) 2019 archive [9]. The ISIC competitions have been a driving force in skin cancer classification research. Professionally annotated and metadata validated by biopsy are presented with digital collections of high-quality images of skin cancer. The goal is to support research that will lead to the development of computer-aided diagnosis CAD methods that can automatically identify melanoma and other kinds of skin cancers. This community also hosts yearly skin lesion challenges in an effort to get more researchers involved in developing better CAD techniques and bringing attention to the rapidly growing problem of skin cancer [13].

**Fig 2:**
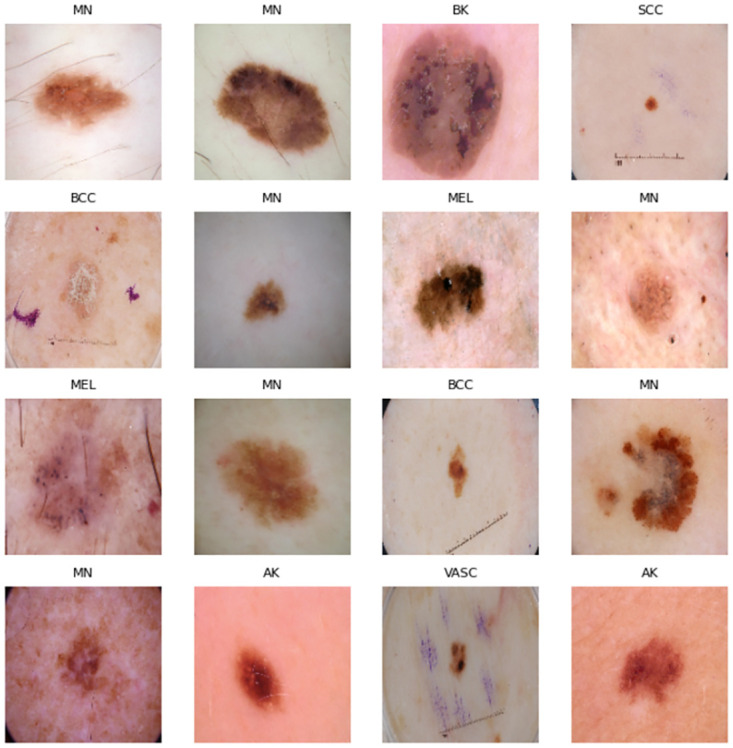
Dermoscopic images of skin cancer disease.

The renowned dataset from the ISIC 2019 archive consists of 25,331 images comprising 8 distinct types of skin cancer: AK, BCC, SCC, BK, DF, MN, MEL, and VASC. This dataset includes the images of the HAM10000 dataset [10], BCN_20000 datasets [11], and MSK Dataset [12] and the images of this dataset obtained in a JPEG format. 46% of image data is collected from female patients, the 54% of image data is collected from male patients. This dataset collects images from patients age range from 5 years to 85 years. Besides the images, the collection of data consists of the corresponding metadata. The meta-data includes the age and gender of the patient, as well as the location of the skin lesion. In this study, we used 440 AK images, 600 BCC images, 800 BK images, 230 DF images, 818 MEL images, 1510 MN images, 245 SCC images, and 200 VASC images, and Fig 2 shows the images of skin cancer.

### 3.2. Data pre‑processing

**Fig 3:**
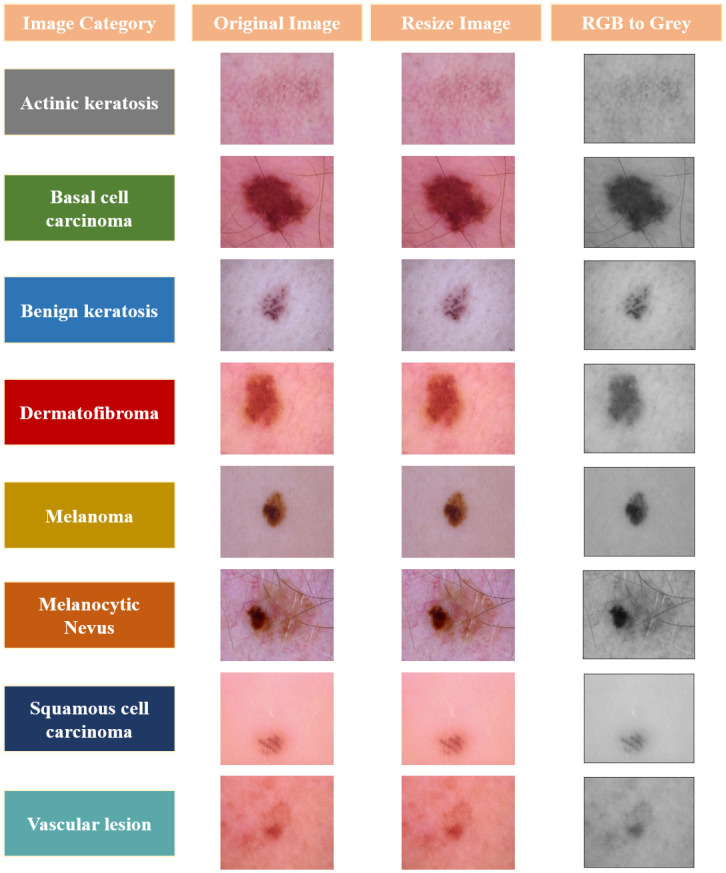
Various phases of data pre-processing.

The processing of images is an essential stage in the process of gathering meaningful data and accurate categorization [58]. A MATLAB tool was used to transform the images from RGB to grayscale before their insertion into the model. Additionally, the resolution of the images was increased to 224 by 224 pixels. Fig 3 illustrates a few instances of images at numerous phases of the pre-processing.

#### 3.2.1. Modified Anisotropic Diffusion Filtering (MADF)

Filtering techniques preserve essential image information while eliminating noise. Filtering algorithms that maintain information are the most efficient at retrieving essential details from a noisy image [52]. During the experimentation phase, the effectiveness of the filter was assessed by applying photos that speckles had impacted as test images. When using an anisotropic diffusion filter, it is possible to preserve and improve edge information while simultaneously reducing noise [59]. The gradient function can detect the data’s noise and edge information. This technique detects variations in the noise gradient outside the edge gradient in images with an extraordinary amount of speckle and very little contrast. Because of these modifications, information from the edges is eliminated more than noise, which results in less accurate filtering [60].

Similarly, due to the image filtering, speckle-reducing anisotropic diffusion cannot preserve all of the border information. OBNLT, which stands for oriented-based non-local techniques, is afflicted with transport noise and cannot retain accurate data. The anisotropic diffusion using the memory-based speckle statistic (ADMSS) provides a more precise depiction [14]. In this study, the MADF has been proposed as a technique for maintaining the precision of image features while simultaneously reducing the amount of image noise and distortion. This filtering method is superior to the others because it can effectively eliminate in-plane multiplicative speckle noise. The suggested method uses association values and noise kurtosis ratios to preserve the data. This process is repeated until the values of the noise component of the image approach those of the Gaussian distribution [61]. In this particular case, the kurtosis needs to be equal to zero. The noise component is represented by Eq (1), and iteration proceeds until the kurtosis of the noise component decreases less than the values calculated by Eq (3). This measurement can be described by Eq (2). The loop terminates when there is even a weak correlation between the image and a noise class. According to the results of Eqs (1) through (7) [15], A and A_0_ represent the genuine image and the noisy image, respectively, while M represents the noise intensity means. By employing Eq (5), one can calculate the kurtosis value k. While Eq (6) computes the intensity correlation of the image, Eq (7) computes the intensity correlation of noise. The proposed filtering generates the most favorable results When ρJ and ρM have the smallest deviation.


$$A_{0\;}=A_{n}$$ (1)

$$n=\frac{A-M}{\sqrt{M}}$$ (2)

$$\mu =\frac{\;\sum_{i=1}^{N}M_{i}}{N}$$ (3)

$$k=\frac{\frac{1}{N}\;\sum_{i=0}^{N}(M-\mu {)}^{4}}{[\frac{1}{N}\;\sum_{i=0}^{N}(M-\mu {)}^{2}{]}^{3}}-3$$ (4)

abs(n-k)≤0.001 (5)

$$\rho_{A}=\frac{\sum_{i=0}^{M-1}\sum_{j=0}^{N-1}i.j.\rho_{A}\left(i,j\right)-\mu A_{x}\mu A_{y}}{\frac{\sum_{i=1}^{N}\left(A_{ix}-\mu_{Ax})(A_{iy}-\mu_{Ay}\right)}{N}}$$ (6)

$$\rho_{M}=\frac{\sum_{i=0}^{M-1}\sum_{j=0}^{N-1}i.j.\rho_{M}\left(i,j\right)-\mu M_{x}\mu M_{y}}{\frac{\sum_{i=1}^{N}\left(M_{ix}-\mu_{Ax})(M_{iy}-\mu_{Ay}\right)}{N}}$$ (7)

**Fig 4:**
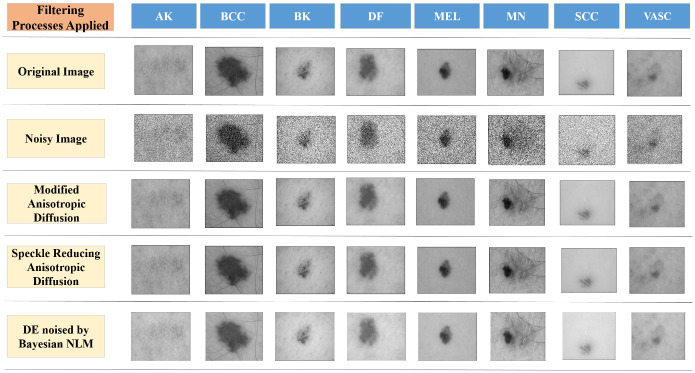
Images of skin cancer eight categories after various diffusion techniques.

Fig 4 depicts the contrast of various Anisotropic Diffusion techniques and an instance of novel pictures. The efficacy of the suggested MADF method for retaining edges was better than that of different methods.

#### 3.2.2. SMOTE TOMEK to equalize the dataset

Two common approaches to resampling are known as over-sampling and under-sampling, respectively. Oversampling leads to an increase in the number of instances belonging to the minority class, whereas under-sampling leads to a drop in the number of instances belonging to the majority class. However, there is also another form of re-sampling technique, which is a combination of the two different ways. In the process of this research study, we made use of a hybrid algorithm called SMOTE TOMEK. It includes the up-sampling method SMOTE and the down-sampling approach TOMEK. TOMEK is an adaptation of condensed nearest neighbors, but SMOTE creates unique data points based on class nearby neighbors. Both methods execute in sequence, and SMOTE picks an instance arbitrarily from the class of minority and then enhances that class by interpolating additional instances into the data. After that, TOMEK will pick a sample arbitrarily and release it if any of its closest neighbors are part of a minority class [30, 62]. SMOTE TOMEK evens out the number of samples of every category in this manner, which allows it to successfully address the issue of dataset inequalities, which is illustrated in Table 2.

**Table 2:** Samples of dermoscopic images before and after SMOTE TOMEK.

No. of Classes	Class Name	No. of Images before up-sampling	No. of Images after up-sampling
0	AK	440	1507
1	BCC	600	1507
2	BK	800	1507
3	DF	230	1510
4	MEL	818	1501
5	MN	1510	1495
6	SCC	245	1509
7	VASC	200	1510

### 3.3. Image segmentation

In dermoscopy images, segmentation is a common technique to distinguish between each pixel. Dermoscopic data on skin cancer are simplified by a segmentation technique designed to reduce their associated computational complexity [63]. The employed segmentation technique enhances the recognition accuracy of the diagnosed picture. When analysis of previously processed data, the research study determined that the preferred outcome was achieved for 2 clusters. Every repetition adjusts the cluster’s center to decrease the gap between the various intensity levels and the cluster’s centroid [16, 64]. By employing Eq (8), the center of the cluster is determined by averaging the intensities of each pixel inside a cluster set. Using Eq (9), the distance is measured.


BK=∑jiN (8)

$$r=\left|B_{k}-a_{i}\right|$$ (9)

In this case, the central point of the kth cluster is denoted by the symbol B_k_, and the intensity within the cluster is represented by j_i_. a_i_ is the notation used to represent the intensity of each pixel at the grey level N. For this experimentation, the clustering approach was utilized to segment the regions. The process of segmenting a sample image is depicted in Algorithm 2.

**Algorithm 2**: Region Segmentation

**N** = number of grey intensity = 256

**B** = Number of centroid = 4

**E** = Enhanced Image

**O** = Segmented output Image

**Start**

 min ← α

  **for** k: 1 → 4

   **for** i: 1→ 256

   *r* ← │B_k_ a_i_—Ea_i_│

   if *r* < min

    j_i_ ← a_i_

    min ← *r*

   **end if**

  **end for**

  B_k_ = ∑ j_i_ / N

 **end for**

 **for** i: 1 → 256

  Oa_i_ ← j_i_

 **end for**

**End**

### 3.4. Feature extractor

In the present study, VGG-19 and Histogram Oriented Gradient were employed independently as feature extraction methods. When these features are extracted by applying HOG and VGG-19, they are combined into a feature vector. This fused vector enables the most precise detection of skin cancer disease from a dermoscopy image.

#### 3.4.1. Histogram Oriented Gradient (HOG)

The results of the experiment indicate that the HOG features exhibit greater reliability when applied to dermoscopy images to detect skin cancer. The HOG feature extractor uses local intensity gradients for data interpretation while processing object data [18].

The image is divided into separate gradients, which are later combined into a single gradient. The segmented image is filtered with a Sobel kernel to determine the gradient direction of M_x_ and M_y_. The magnitude of each pixel’s angle and gradient are computed using Eqs (10) and (11), respectively.


$$f_{\left|M(i,j)\right|}=\sqrt{M_{x}(i,j{)}^{2}+M_{y}(i,j{)}^{2}}$$ (10)

$$\theta_{\left|M(i,j)\right|}={\text{tan}}^{-1}(\frac{M_{y}(i,j)}{M_{x}(i,j)})$$ (11)

Here, f represents the magnitude of the gradient in path θ for the image identified by row i and column j. By employing the gradient and angle, a histogram is determined. Each histogram block is utilized to construct the normalization vector. In the end, Eq (12) reveals the HOG feature identifier using eight block sizes.


$$V=\sum_{i}^{N}\frac{V_{i}}{\sqrt{v_{i}^{2}+k}}$$ (12)

The standardized vector is merged into an individual block, and Eq (12) is used to produce the HOG feature vector. It distinguishes the essential area of an image with valuable data by creating a histogram and omitting the irrelevant data. This is a powerful feature creator for image object identification. It is significantly simpler and faster to perform computations. In addition, HOG explains an entire image or region, while SIFT [19] and other extraction of feature methods define a particular point within an image [65]. Furthermore, it gives more reliable features compared to various feature extraction approaches.

For HOG extraction of features, 64 × 128-pixel images are pre-processed. This is achievable with 8 × 8 and 16 × 16 image patches, respectively. After that, the gradient is estimated for every pixel in both the x and y directions. The value of each pixel in each region can be determined more accurately with the assistance of a gradient approach. The same procedure is replicated for each pixel within the image.

Consequently, proceed to the entire image; the features are stored for each fewer image section. This experiment produces a 9 × 1 matrix for each unit while splitting the picture into 88% units. Finally, four 8 × 8 units combine to create a 16 × 16 block. Because the histogram of every single 8 x 8 unit carries out a 9 × 1 structure, the 16 × 16 block as a whole is made up of a single vector that is 36 × 1. In Eq (5), the size of the normalized vector is 36 × 1. The horizontal gradient of this standardized variable is seven and the vertical gradient is fifteen, resulting in a value of 7 × 15 = 105. According to this study, the entire number of characteristics for each photo by employing the HOG technique is 105 × 36 × 1 = 3780.

#### 3.4.2. A feature extractor and classifier based on VGG19

VGG19-based extraction of image features is one of the major developments in computer vision (CV). In this study, generic CNN and pre-trained VGG19 were evaluated. The generic model works poorly with small datasets, while the pre-trained model produces effective results. We fine-tune a pre-trained VGG-19 model as a feature extractor by applying our experiment dataset. This network model was created using the 19-layer variant of VGGNet. In this experiment, VGG-19 beat VGG-16 and other DL classifiers, such as AlexNet, ResNet-50, and Inception-V3 models as mentioned in Table 2.

**Fig 5:**
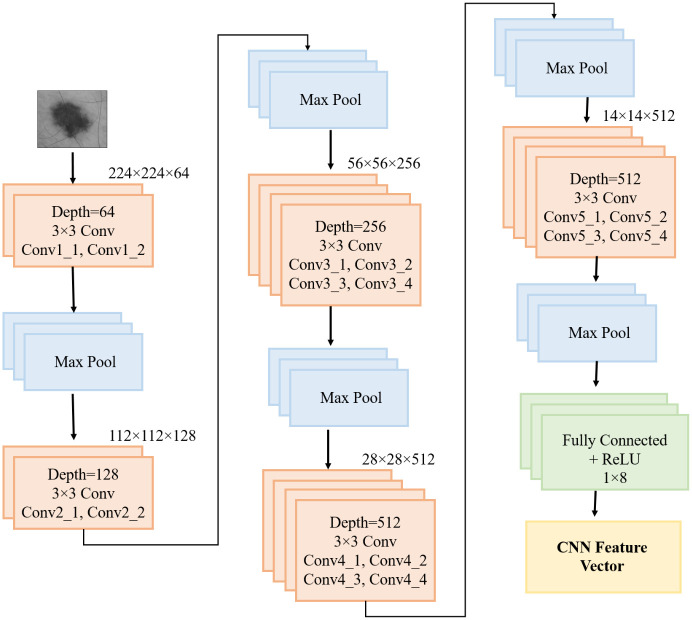
Overall structure of the VGG-19 model.

Fig 5 depicts that the VGG-19 model encompasses 16 convolution layers and then 3 fully connected layers (FCLs). As the activation function for every layer of convolution results, a ReLU function is utilized. Five sequential layers of Max-Pooling subdivide the entire convolution portions into 5 subsections. The 1^st^ and 2^nd^ subsections comprise 64 and 128 convolution layers, respectively.

With values ranging from 256 to 512, four convolution layers are used to construct the following three subsections. Subsections of the convolutional layer are followed by pooling layers to reduce the trainable parameter. The last layer of our VGG-19 model is replaced with the SoftMax classification Function. Two hidden layers containing 4096 and 512 units are present before the SoftMax output function. L2 regularization was applied to all FCLs except the dropout layer to prevent overfitting during the fine-tuning procedure.

Data fusion was utilized in a variety of machine learning and CV tasks in the literature [51]. The number of feature vectors generated by two feature extractors is 14,096 and 13,780, respectively. Eqs (13) and (14) are used to derive the VGG19 and HOG features. Eq 15 is a notation for the process of combining the features that were extracted into a single vector.


$$f_{HOG1xn}=\{{HOG}_{1x1},{HOG}_{1x2},{HOG}_{1x3},{HOG}_{1xn}$$ (13)

$$f_{CNN1xm}=\{{CNN}_{1x1},{CNN}_{1x2},{CNN}_{1x3},{CNN}_{1xm}$$ (14)

$$fused{\left(featuresvector\right)}_{1xq}=\sum_{i=1}^{3}\{f_{HOG1xn},f_{CNN1xm}\}$$ (15)

The proposed approach uses a feature fusion that relies on entropy, which works with an attached direction with 1 × 1186 dimensions [66]. The feature vector has been given entropy to select the ideal characteristics according to score values. Both (16) and (17) are equations that characterize the features of probability, and (16) and (17) describe entropy. Eq (17) was utilized to mathematically demonstrate the process of selection of features [20, 64]. Entropy was used to select 1186 score-based characteristics among 7876 features. Classifiers were supplied with the final features applied to identify skin cancer images.


$$B_{Se}=-NSe_{b}\sum_{i=1}^{n}p(f)$$ (16)

$$F_{Select}=B_{Se}(\text{max}\left(f_{i},1186\right))$$ (17)

In Eqs (16) and (17), the symbol p denotes the feature’s possibility, while the symbol *Se* represents entropy. The selected features are presented to the classifiers at the end of the process. On a fusion of HOG and DL characteristics, the suggested approach was evaluated on the direction of a feature. There are sixteen layers of CNN, 3 FCLs, and one SoftMax activation included in the VGG-19 architecture [24]. The FCL and final layers of all network designs are similar. Max pooling was conducted with stride 2 within the window size. The first two layers of the three FCLs produce a total of 4096 features, while the third layer provides 1000 channels. The final layer contains eight neurons, indicating the outcome of skin cancer disease.

## 4. Experimental results

To evaluate the results of the computational analysis carried out in this study, the parameters of accuracy (ACC), recall (REC), precision (PREC) and F1-score were utilized. The confusion matrix is used to derive these parameters by using the terms “True Positive,” “False Positive,” “True Negative,” and “False Negative,” respectively. The formulas for computing the rate of the confusion matrix for these 4-evaluation metrics are as follows:

$$ACC=\frac{TP+TF}{TP+FN+FP+TN}$$ (18)

$$REC=\frac{TP}{TP+FN}$$ (19)

$$PREC=\frac{TP}{TP+FP}$$ (20)

$$F1-score=2\times (\frac{Precision\times Recall}{Precision+Recall})$$ (21)

Experiments were performed by employing a locally based machine platform that fulfilled all requirements. The ISIC 2019 dataset is divided into the ratio of 70:20:10. The 70% of images are used for training, 20% for testing and 10% for validation. Multiple DL model hyper-parameters were utilized to train the suggested DVFNet architecture. The total trainable parameters employed by the DVFNet is 1,126,439. Using a 0.001 learning rate, a batch size of 32 and 40 epochs were used with the RMSprop optimizer. In the present study, the SoftMax function was utilized during model training.

Feature extraction is a necessary step to execute accurate categorization of the skin cancer disease. This preliminary evaluation assists in identifying the best CNN model for extracting features. VGG-19 works better compared to other CNN classifiers such as Inception-V3, AlexNet, ResNet-50, and VGG-16 when executing on the test and training data as a pre-trained classifier. Table 3 presents a comparison of many CNN pre-trained classifiers using experimental data. When training and evaluating their respective models, all CNN classifiers use the same dataset as a benchmark.

**Table 3:** Comparison of the results obtained by different CNN models.

Classifiers	Accuracy	F1-score	Recall	Precision
AlexNet	85.44%	85.81%	82.12%	88.74%
VGG-16	89.49%	89.28%	89.13%	90.37%
Inception-V3	88.94%	88.70%	88.47%	89.55%
ResNet-50	83.41%	83.71%	80.37%	87.29%
Proposed DVFNet without SMOTE TOMEK	77.97%	78.19%	69.49%	85.48%
Proposed DVFNet with SMOTE TOMEK	98.32%	98.19%	98.23%	98.23%

### 4.1. Accuracy and loss of proposed DVFNet

**Fig 6:**
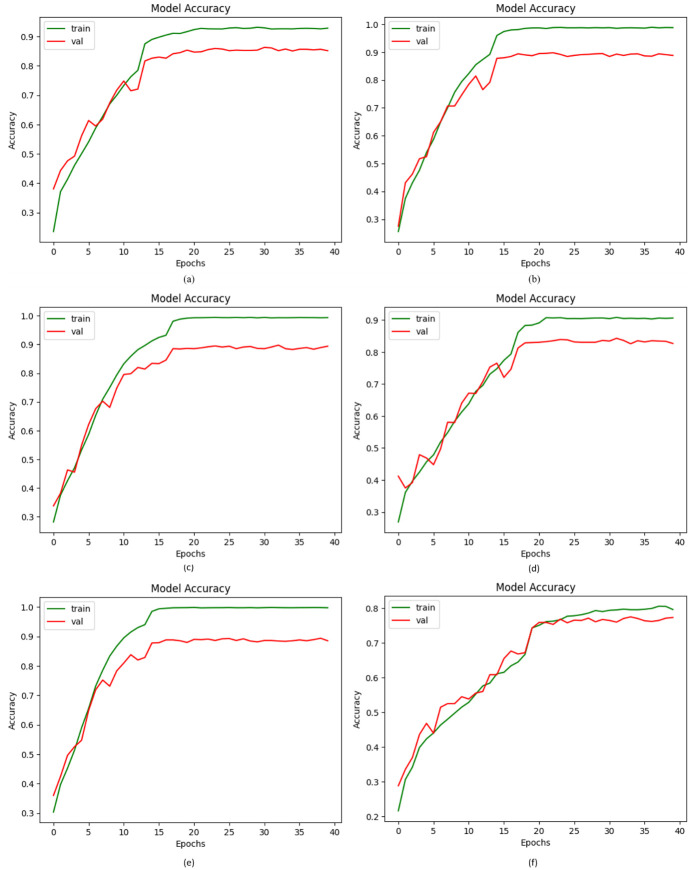
Accuracy of the Proposed DVFNet model with other CNN classifiers; (a) AlexNet, (b) VGG-16, (c) Inception-V3, (d) ResNet-50, (e) Proposed DVFNet using SMOTE TOMEK, (f) Proposed DVFNet prior to SMOTE TOMEK.

The term “accuracy" refers to the overall accuracy of the structure, defined as the proportion of total samples accurately identified by the classifier. Using a similar dataset and up-sampling, we evaluated our proposed DVFNet and current CNN classifiers, such as AlexNet, Inception-V3, VGG-16, and ReNet-50. Furthermore, we contrasted the suggested DVFNet prior to implementing up-sampling. The system comprising up-sampling yields outstanding outcomes for the suggested approach. Fig 6 depicts the enormous enhancement the suggested DVFNet architecture achieves through the up-sampling strategy.

**Fig 7:**
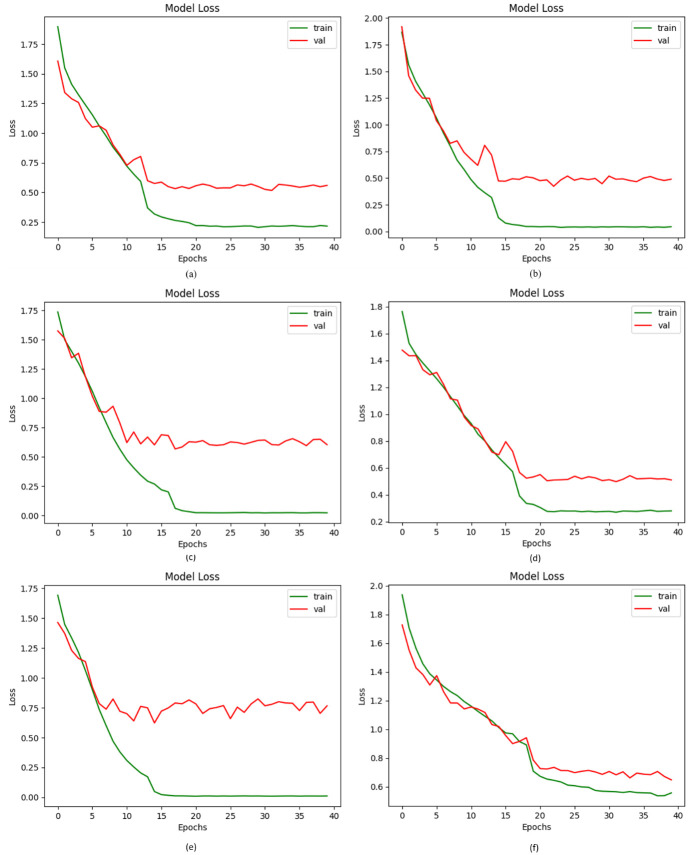
Loss values of the Suggested DVFNet with other CNN classifiers; (a) AlexNet, (b) VGG-16, (c) Inception-V3, (d) ResNet-50, (e) Proposed DVFNet using SMOTE TOMEK, (f) Proposed DVFNet without SMOTE TOMEK.

In addition, loss functions are developed to measure the overall amount of estimation required for determining the estimated value from the actual value [67]. To calculate the amount of loss acquired as a consequence of our study, we picked to use the categorical cross entropy method, as presented in Fig 7. Cross entropy loss is the parameter employed by DL for assessing the effectiveness of a classification model [68]. Using the following equation, we can calculate the loss:

$$Loss=y-\bar{y}$$ (22)

$$L_{CE}=-\sum_{n=1}^{k}\left(L_{i}\;\text{log}{(p}_{i})\right)$$ (23)

### 4.2. AUC of proposed DVFNet against others CNN models

**Fig 8:**
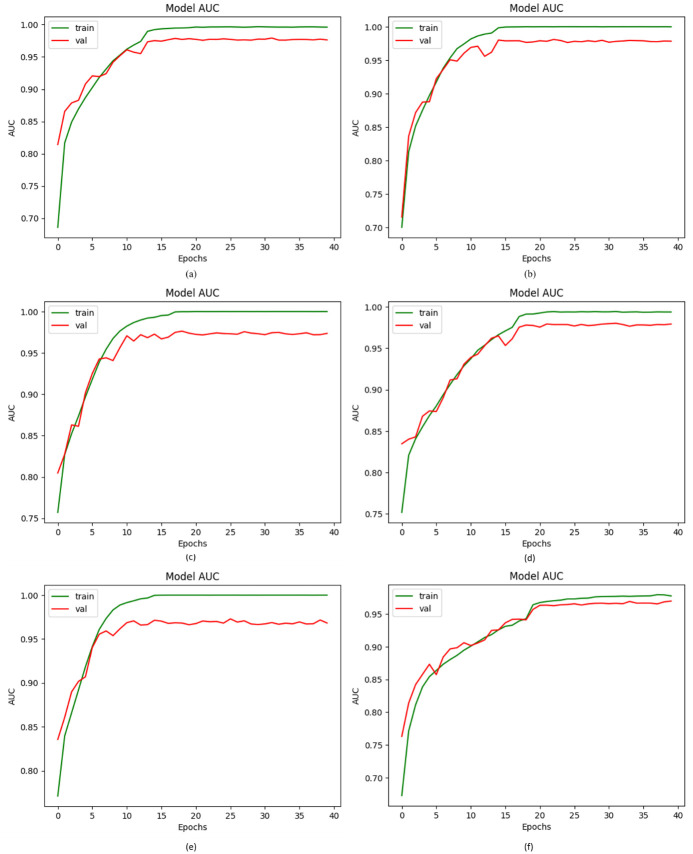
AUC values of the Proposed DVFNet model with other CNN classifiers; (a) AlexNet, (b) VGG-16, (c) Inception-V3, (d) ResNet-50, (e) Proposed DVFNet using SMOTE TOMEK, (f) Proposed DVFNet prior to SMOTE TOMEK.

In classification analysis, Area Under the Curve (AUC) is employed to assess which of the utilized models best predicts the classes. In simple terms, the AUC evaluates the performance of the classifier across all potential thresholds. A classifier’s AUC corresponds to the probability that it will put a randomly selected positive instance above a randomly selected negative instance. To demonstrate the efficacy of the proposed DVFNet model, we compared its outcomes to those of four CNN methods of classification working as a baseline. The AUC values for the four CNN methods of classification such as AlexNet, VGG-16, Inception-V3, and ResNet-50 were 98.12%, 97.88%, 97.23%, and 98.12%, respectively. As shown in Fig 8, the proposed DVFNet model that included up-sampling produced an AUC of 98.90%, while the suggested DVFNet model that did not include up-sampling achieved an AUC of 96.99%.

After examining the findings of the previous evaluation, we’ve found that the AUC values obtained from the proposed DVFNet model are superior to those provided by other CNN classifiers.

### 4.3. Confusion matrix of proposed DVFNet

**Fig 9:**
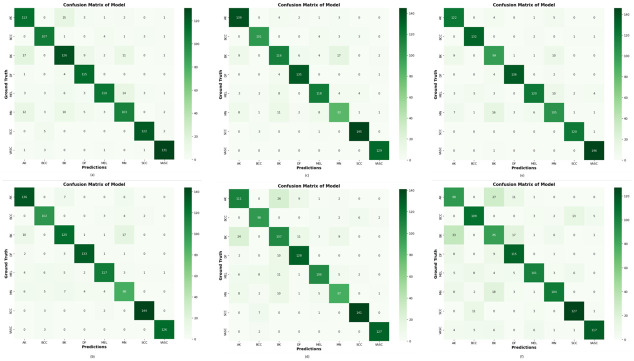
Confusion Matrix of the Suggested DVFNet with others CNN classifier; (a) AlexNet, (b) VGG-16, (c) Inception-V3, (d) ResNet-50, (e) Proposed DVFNet using SMOTE TOMEK, (f) Proposed DVFNet without SMOTE TOMEK.

In order to demonstrate that the DVFNet model that we proposed is accurate utilizing a confusion matrix, we compared it to four other CNN models such as AlexNet, VGG-16, Inception-V3, and ResNet-50. As seen in Fig 9, the usage of SMOTE TOMEK leads to considerable advancements for the DVFNet model.

**Fig 10:**
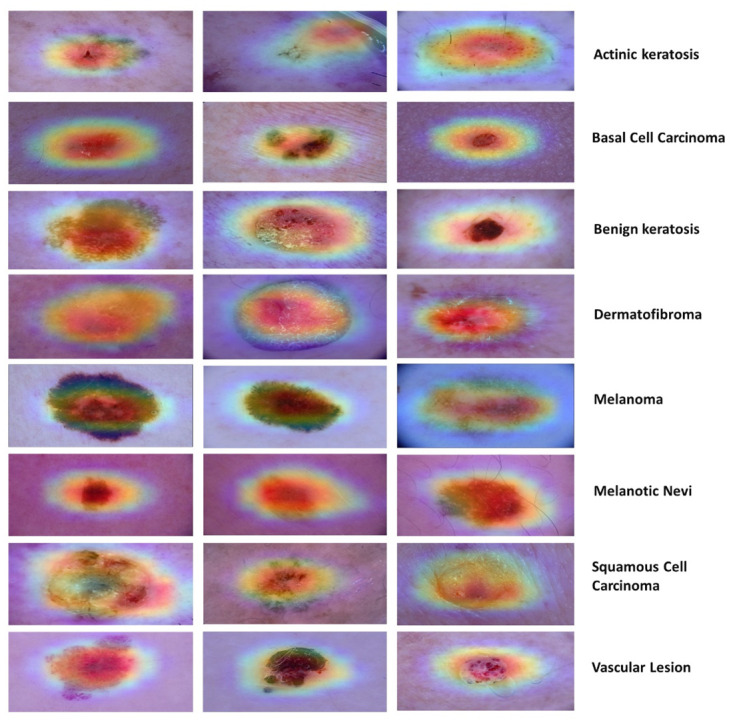
Assessment with grad-CAM of the proposed model for skin cancer diagnoses.

Additionally, the output of our proposed DVFNet model was represented through the use of a Grad-CAM heat map. The function of the heat map is to identify the specific region of the epidermal layer affected by the disease [69]. The heat map for the DVFNet model that has been proposed is found in Fig 10.

### 4.4. Comparison of the proposed DVFNet using state-of-the-art

Our proposed DVFNet model is contrasted with the current studies [21–30]. Furthermore, the suggested model is contrasted using the outcomes of these [23–30] studies. Table 4 contains an in-depth examination of the suggested DVFNet in terms of several different assessments of evaluation metrics, i.e., accuracy, F1-score, precision, and recall, in contrast to modern state-of-the-art research.

**Table 4:** List of relevant studies using DL and ML algorithms for skin cancer identification.

Ref	Year	Datasets	Model	Accuracy	Precision	F1-score	Recall
[21]	2022	HAM10000	DenseNet-169	92.25%	92.95%	93.27%,	93.59
[22]	2022	ISIC 2020	DCNN	90.42	90.48%	90.41%	90.39%
[23]	2022	ISIC 2019	GoogleNet	73%	82%	81%	80%
[24]	2023	ISBI 2016	DenseNet-201 and MobileNet with SVM	88.02%	88.24%	93.10%	97.54%
[25]	2022	HAM10000	EfficientNet-B4	88%	88%	87%	88%
[28]	2022	HAM10000	CNN	86%	84%	86%	86%
[29]	2023	ISIC 2018	Inception-V3	91.26%	89%	92%	92%
[30]	2023	ISIC 2020, Derm-IS, HAM10000	DSCC_Net	94.17%	93.76%	93.93%	94.28%
[70]	2023	ISIC 2019	AlexNet-GoogLeNet-VGG16-ANN	96.10%	88.69%	88.90%	——
[71]	2023	HAM 10000	Multiscale Attention CNN	91.60%	96.40%	73.50%	——
**Ours**	-	ISIC 2019	Proposed DVFNet with SMOTE TOMEK,	98.32%	98.23%	98.19%	98.23%

### 4.5. Statistical analysis of the proposed DVFNet using ANOVA test

This study used statistical hypothesis testing utilizing ANOVA to assess the efficacy of each of the methods [72]. The DVFNET model’s mean accuracy is comparable with other techniques, according to the null hypothesis (H0). In contrast to the null hypothesis (H0), Ha predicts that at least one model has a different mean. The results indicate a significance level of p = 1.0580310 and an F-statistic of 90.1392x10-14. As the p-value is below 0.05, we agree with the competing hypothesis that the DVFNET method provides more reliable results. The probability of the F statistic is then determined using the F-test to understand whether it’s below or equal to the significance level. In such a case, we should accept the null hypothesis; otherwise, we reject it. The results of the ANOVA show that DVFNet has a much higher F-statistic. As the F-statistic rises, the evidence for a significant difference in mean scores between the groups increases.

### 4.6. Discussions

The identification of skin cancer is a complex procedure. A skilled dermatologist makes a diagnosis through a series of steps, starting with the naked eye identifying abnormal cancerous cells, followed by the dermoscopy that employs an optical lens for analyzing abnormalities in extreme detail, the biopsy is performed [31–38, 73]. Skin cancer is a significant concern that demands quick evaluation from medical professionals [74, 75]. Most individuals do not regularly consult their dermatologist, resulting in fatally delayed treatments. The manual evaluation of skin conditions is highly challenging and distressing for the individual [39]. The circumstances in which there is a lack of professionals or medical skills, a computer-aided system assists health professionals in diagnosing skin cancer [40]. As a direct consequence of this, we created a CNN-based DVFNet model that can identify a wide variety of skin conditions correctly. The results showed that DVFNet outperforms the VGG-16, AlexNet, ResNet-50, and Inception-V3 as mentioned in Table 3.

The DVFNet works better than other models because it extracts the features separately by utilizing HOG and VGG16. The prominent features are then combined to form a feature vector provided to CNN for classification. With the help of this model, dermatologists can begin treating their patients for MEL, MN, AK, BK, DF, BCC, VASC, and SCC at an early stage. Kousis et al. [21] proposed 11 distinct CNN architecture candidates. Eleven different CNN architectures were trained and tested across seven different types of skin cancer using the HAM10000 dataset. DenseNet169 produced the most impressive results out of the 11 CNN architecture variants. A lightweight and less expensive DCNN technique was offered by Kaur et al. [22] for accurately identifying melanoma, a kind of skin cancer. For their research, dermoscopy photos from the ISIC 2016, 2017, and 2020 containing various cancer samples were collected. When applied to the ISIC data sets from 2016, 2017, and 2020, respectively, the proposed model achieved an accuracy of 81.45%, 88.24%, and 90.41%. Aljohani et al. [23] evaluated multiple CNN architectures, such as MobileNet-V2, DenseNet-201, ResNet50-V2, Xception, ResNet152-V2, VGG-16, GoogleNet, and VGG-19, as well as the associated models for DL on GPUs. A dataset comprising 7146 images was analyzed with their classifiers, and the results were compared. The results demonstrated that GoogleNet attained the highest performance among other classifiers. Keerthana et al. [24] introduced two innovative CNN hybrid approaches using an SVM classifier at the final layer for categorizing dermoscopy images into benign or melanoma tumors. The characteristics taken by the first and second CNN models were combined and passed to the SVM classifier. Using the ISBI 2016 dataset, their models obtained an accuracy of 88.02% and 87.43%.

Ali et al. [25] uses the HAM10000 dataset to analyze the performance of EfficientNets B0-B7 models. This method assessed and compared the effectiveness of each version of EfficientNet on a multiclass classification problem with unequal class representations. EfficientNet-B4 and B5 Models with an intermediate level of complexity provide the best performance results. Alwakid et al. [28] created the DL method to extract prominent features from the infected area of skin. To improve the image’s quality at first, ESRGAN was employed. Next, segmentation was used to divide the image into Regions of Interest (ROI). To fix the data inconsistency, this method utilized data augmentation technique. The image was subsequently classified using a CNN and an altered variant of Resnet-50. Lembhe et al. [29] used an artificial method for detecting skin cancer using image processing and ML. Image super-resolution (ISR) approaches produce high-resolution images or series from LR images. To improve the accuracy of the CNN model, a technique employing deep learning on ISR was used. Tahir et al. [30] proposed an innovative CNN-based approach, DSCC_Net, and examined it using three freely accessible benchmark databases (i.e., HAM10000, ISIC 2020, and DermIS). Their proposed model was successful in classifying MEL, BCC, MN, and SCC with an accuracy of 94.17%, 94.28% precision, recall of 93.76%, and 93.93% F1-score. Olayah et al. [70] classified skin cancer utilizing a hybrid deep-learning approach. An impressive 96.10% accuracy is achieved by the hybrid model when applied to the ISIC 2019 dataset. However, Qain et al. [71] suggested a deep convolutional neural network for dermoscopy image classification, using class-specific loss weighting and multi-scale attention blocks. The HAM10000 dataset is employed to assess the performance of the proposed model. This algorithm has a 91.6% accuracy rate for skin cancer identification.

Table 4 displays a comparison between the DVFNet model and the SOTA classifiers concerning their respective classification accuracies. The model can identify MEL, MN, AK, BK, DF, BCC, VASC, and SCC from dermoscopy images. When comparing experimental results with contemporary SOTA methods, it is clear that this has contributed significantly to aiding the clinical expert. The Results showed that DVFNet effectively extracts prominent and discriminative features from the images with an accuracy of 98.32%. The classification performance of pre-trained models are negatively impacted by the initial stage of their process, which involves reducing the deep networks to their final ConvLs. A large number of input neurons and the small size of the filter of these pre-trained classifiers are unable to capture important features. The DVFNet offered the solution to these problems. This method effectively extracts discriminative features using dermoscopy images to diagnose multiple types of skin cancer. DVFNet enhances convergence and classification efficacy and substantially eliminates the adverse effects of structured noise. DVFNet utilizes the up-sampling technique to achieve outstanding results. The Grad-CAM is used to illustrate the area of the skin that is infected, as shown in Fig 10. DVFNet correctly classifies MEL, MN, AK, BK, DF, BCC, VASC, and SCC using dermoscopy images and aids dermatologists.

## 5. Limitation of the work

Our proposed technique is evaluated using a dataset including class imbalance data. We increased the number of images using borderline SMOTE, but testing the proposed model on a more comprehensive dataset is better. The data available in the real world often vary from the data found in public datasets. The real-world dataset is needed to test the real potential of the proposed method.

## 6. Conclusion

Early detection of skin cancer allows for efficient treatment and prevention. Multiple DL methods have been reported for successfully identifying skin cancer. Unfortunately, these models are still ineffective at classifying cancer due to the shortage of images representing dangerous skin lesions. The studies illustrated that with deep learning image analysis, healthcare professionals can more accurately diagnose skin cancer using dermoscopy images. To train and test our model, we utilized the ISIC 2019 dataset to diagnose skin cancer. We have developed MADF, which retains edges considerably better than existing methods and reduces noise from resized grayscale dermoscopy images. In this study, the most effective feature extractor is proposed by the combination of HOG and VGG19. To get significant results, the CNN classifier uses these fused feature vectors for the multiclassification of skin cancer. It is difficult to compare results correctly since different researchers employed various datasets, analytical techniques, and computing resources. A table was made to show the similarities and differences between our suggested model and other models. Table 4 compares the effectiveness of several techniques for detecting skin cancer using dermoscopy images. The DVFNet achieves the highest accuracy of 98.32% compared to the baseline and state-of-the-art models. In future work, we will employ federated learning with a proposed model to achieve better results.
